# Facility-Level Availability of Japanese Society of Medical Oncology Specialists and Recorded First-Line Treatment-Process Duration in Pancreatic Cancer: A Nationwide Center for Cancer Genomics and Advanced Therapeutics Registry Analysis

**DOI:** 10.3390/curroncol33070393

**Published:** 2026-07-01

**Authors:** Shinya Kajiura, Hironaga Satake, Naohiko Nakamura, Ryuji Hayashi

**Affiliations:** 1 Department of Medical Oncology and Palliative Medicine, Toyama University Hospital, Toyama 930-0194, Japan; naohiko@med.u-toyama.ac.jp (N.N.); hsayaka@med.u-toyama.ac.jp (R.H.); 2 JSMO Secretariat Division, Japanese Society of Medical Oncology, Tokyo 105-0013, Japan; satakeh@kochi-u.ac.jp; 3 Department of Medical Oncology, Kochi Medical School, Kochi University, Nankoku 783-8505, Japan

**Keywords:** pancreatic cancer, Japanese Society of Medical Oncology, medical oncology specialist, Center for Cancer Genomics and Advanced Therapeutics, C-CAT, real-world data, treatment-process endpoint, registry-based study

## Abstract

Facility-level availability of Japanese Society of Medical Oncology (JSMO) specialists may influence care processes, but national cancer genomic medicine data rarely capture patient-level specialist involvement. We conducted a nationwide retrospective analysis of pancreatic cancer cases in the Center for Cancer Genomics and Advanced Therapeutics (C-CAT). The primary exposure was facility-level registry-listed JSMO specialist count (0–1 vs. ≥2 specialists), with ≥2 interpreted as a proxy for minimum plural specialist-team availability. The primary endpoint was time from systemic therapy start to recorded first-line treatment end. The primary cohort included 14,568 patients at 261 facilities. Median recorded first-line treatment-process duration was 5.7 months in the 0–1 specialist group and 6.4 months in the ≥2 specialist group. In the clinical plus facility-adjusted Cox model, ≥2 specialist availability was associated with a lower hazard of recorded first-line treatment end (HR 0.895, 95% CI 0.810–0.988; *p* = 0.028). Supportive overall survival findings did not indicate a survival advantage, reinforcing the operational nature of the primary endpoint. These findings indicate a facility-level association with an operational treatment-process endpoint, not patient-level specialist involvement, treatment efficacy, survival benefit, facility ranking, or causality. Chemotherapy-specific national database elements are needed to evaluate specialist contribution directly.

## Simple Summary

Pancreatic cancer treatment often requires coordinated systemic therapy, but national data rarely show how specialist-team availability relates to treatment processes. We used nationwide data from the Center for Cancer Genomics and Advanced Therapeutics (C-CAT) to compare facilities with 0–1 versus ≥2 registry-listed Japanese Society of Medical Oncology (JSMO) specialists. The analysis focused on time from systemic therapy start to recorded first-line treatment end. Among 14,568 patients at 261 facilities, median recorded first-line treatment-process duration was 5.7 months at facilities with 0–1 specialists and 6.4 months at facilities with ≥2 specialists; the association persisted after clinical and facility adjustment. Supportive overall survival did not support a corresponding survival advantage. These results show a facility-level association in registry-derived treatment-process data, not direct specialist involvement, treatment efficacy, facility quality, causality, or survival benefit. Chemotherapy-specific national database elements are needed for direct evaluation.

## 1. Introduction

Pancreatic cancer remains a clinically difficult malignancy in which systemic therapy is required for many patients with advanced, recurrent, or unresectable disease. Contemporary management requires coordinated decision-making across treatment sequencing, symptom burden, toxicity management, nutritional status, performance status, biliary or gastrointestinal complications, and timely transition of care [1,2,3]. In real-world pancreatic cancer practice, recorded first-line treatment duration can be shaped by disease aggressiveness, tolerability, clinical deterioration, patient preference, access to subsequent therapy, referral pathways, and documentation practices [4,5,6]. Therefore, a record-derived treatment-process endpoint can be clinically and operationally informative, provided that it is not interpreted as progression-free survival, treatment efficacy, survival benefit, or quality of care itself [7].

The increasing complexity of systemic anticancer therapy has heightened the importance of medical oncology expertise and team-based care infrastructure. In Japan, Japanese Society of Medical Oncology (JSMO)-certified specialists represent a national specialist workforce supporting systemic therapy delivery, and medical oncology departments have expanded their responsibilities within designated cancer care hospitals, including drug therapies for pancreatic cancer and newer therapeutic modalities [8]. In facility-level registry research, registry-listed specialist count can be examined as one measurable proxy for oncology care-delivery infrastructure, alongside multidisciplinary coordination, genomic medicine functions, clinical trial access, and treatment documentation capacity.

The Center for Cancer Genomics and Advanced Therapeutics (C-CAT) provides a national framework for cancer genomic medicine data in Japan. Since comprehensive genomic profiling was introduced under the national health insurance system, C-CAT has aggregated clinical and genomic information generated through the Japanese cancer genomic medicine network and has supported secondary use of nationwide real-world oncology data [9,10,11]. Because C-CAT is a cancer genomic medicine resource rather than a chemotherapy-specific clinical database, its treatment fields are best suited to operational, record-derived process measures when data boundaries are explicitly reported [12].

We therefore conducted a nationwide retrospective C-CAT registry-based facility-level analysis of pancreatic cancer to examine the association between facility-level registry-listed JSMO specialist availability and recorded first-line treatment-process duration. The primary exposure was facility-level registry-listed JSMO specialist count, categorized as 0–1 versus ≥2 specialists, with ≥2 specialists interpreted as an operational proxy for minimum plural specialist-team availability. The primary endpoint was time from systemic therapy start to recorded first-line treatment end. We also evaluated supportive overall survival, a PAAD-only sensitivity analysis, endpoint-missingness sensitivity analyses, and denominator construction. The objective was to evaluate whether facility-level registry-listed JSMO specialist availability is associated with this operational treatment-process endpoint while maintaining interpretation at the facility and record-derived endpoint level.

## 2. Materials and Methods

### 2.1. Study Design and Data Source

We conducted a nationwide retrospective registry-based facility-level analysis using pancreatic cancer data from the Center for Cancer Genomics and Advanced Therapeutics (C-CAT), a national cancer genomic medicine data resource in Japan that includes patients who underwent comprehensive genomic profiling under the Japanese cancer genomic medicine system. Patient-level C-CAT clinical data were linked to validated facility-level registry-listed Japanese Society of Medical Oncology (JSMO) specialist variables using facility identifiers. The exposure was assigned at the treating-facility level, whereas outcomes were constructed at the patient level from available C-CAT clinical data fields. This study was intended to evaluate facility-level associations and data-system feasibility, not direct patient-level JSMO specialist involvement, treatment efficacy, facility ranking, or causal effects.

### 2.2. Study Cohort

Eligible cases were definite pancreatic cancer cases registered in C-CAT with a facility identifier available and linkable to the validated facility-level JSMO variables. Clinical source data were derived from the two pancreatic cancer case-background datasets, pancreas_case_batch_01 and pancreas_case_batch_02. Mutation files were not used as the main clinical source data for cohort construction or endpoint derivation.

For the primary first-line time-to-event analysis, patients were required to have a reconstructed systemic therapy start date, a recorded first-line treatment end date or censoring date, and a non-negative interval from systemic therapy start to event or censoring. Pancreatic subtype was categorized as PAAD, other/non-adenocarcinoma, or missing/unspecified subtype; missing/unspecified subtype was not assumed to be PAAD. A PAAD-only analysis was conducted as a sensitivity analysis. A broader endpoint-input cohort was used to assess endpoint-input availability and denominator construction. Endpoint-specific cohorts were defined for supportive overall survival and for the secondary/exploratory analysis of time from systemic therapy start to recorded second-line treatment end, using the corresponding event or censoring dates and non-negative interval requirements.

### 2.3. Facility-Level JSMO Specialist Availability

The primary exposure was facility-level registry-listed JSMO specialist count, categorized as 0–1 versus ≥2 specialists. The cutoff of ≥2 specialists was selected a priori as an operational proxy for minimum plural specialist-team availability, distinguishing facilities with at least minimal plural specialist-team availability from those with no or only single listed specialist availability. This threshold was not intended to model a linear dose-response relationship between specialist count and treatment-process duration and was not selected by statistical optimization. This variable was interpreted strictly as a facility-level proxy and did not indicate patient-level treating-physician certification or direct JSMO specialist involvement.

### 2.4. Genome Medicine Facility Category

Genome medicine facility categories were grouped into three levels for analysis: genome medicine cooperative hospital, genome medicine hub hospital, and genome medicine core hospital. These categories were treated as broad facility-level background categories within the Japanese cancer genomic medicine delivery system. The primary research question concerned facility-level registry-listed JSMO specialist availability and recorded first-line treatment-process duration, not detailed evaluation of the cancer genomic medicine delivery system or ranking of genome medicine facility hierarchy. Therefore, subcategories within cooperative hospitals were not modeled separately. The three-level genome medicine facility category was used as a facility-level adjustment variable in Cox models.

### 2.5. Endpoints

#### 2.5.1. Primary Endpoint

The primary endpoint was time from systemic therapy start to recorded first-line treatment end. Time zero was the reconstructed systemic therapy start date, and the event was the recorded first-line treatment end date. Patients without a recorded first-line treatment end date were censored at the death date or last survival confirmation date when available. Negative intervals were excluded. This endpoint was constructed as a recorded first-line treatment-process duration derived from available C-CAT treatment records and was not defined as progression-free survival. Endpoint ascertainment was evaluated descriptively. Two endpoint-sensitivity analyses were performed: a complete recorded-end-date analysis and an extreme event-at-censor analysis in which censored observations in the primary cohort were treated as events. Recorded 1L end-date availability was also modeled as a data availability outcome.

#### 2.5.2. Supportive Overall Survival

Supportive overall survival was defined as time from systemic therapy start to death. Patients without a recorded death date were censored at the last survival confirmation date. Overall survival was included for supportive clinical context only and was not used as the primary basis for evaluating specialist-team availability.

#### 2.5.3. Secondary/Exploratory Second-Line Endpoint Definition

The secondary/exploratory endpoint was time from systemic therapy start to recorded second-line treatment end. The event was the recorded second-line treatment end date. Patients without a recorded second-line treatment end date were censored at the death date or last survival confirmation date when available. This endpoint was included for exploratory and hypothesis-generating comparison only and was interpreted as a recorded treatment-process endpoint rather than treatment efficacy, survival benefit, or patient-level specialist involvement.

### 2.6. Covariates

Covariates used in the adjusted Cox models were age, treatment start year, sex, Eastern Cooperative Oncology Group performance status (ECOG PS), specimen type, pancreatic subtype group, and the three-level genome medicine facility category. ECOG PS 2, 3, and 4 were collapsed into the ECOG PS ≥2 category. Fresh frozen and other specimen types were collapsed into Other. Nonblank non-PAAD pancreatic subtypes were collapsed into Other/non-adenocarcinoma, and missing or unspecified pancreatic subtype was retained as a separate missing/unspecified category rather than being assumed to be PAAD. Missing/unknown ECOG PS and missing/unspecified pancreatic subtype were retained as explicit categories in adjusted models. Stage before first treatment was not included in the primary adjusted models because missing or unknown values were frequent. Multiple imputation was not used because missingness included registry-derived unknown/unspecified categories and structurally unavailable information. During construction of the final analysis dataset, we reviewed available C-CAT source fields; however, treatment regimen, FOLFIRINOX, gemcitabine/nab-paclitaxel, dose intensity, toxicity, progression, discontinuation reason, resection status, and metastatic burden could not be reliably derived as structured analysis variables and therefore were not included as covariates. No additional covariates beyond those used in the final Cox model table were introduced.

### 2.7. Statistical Analysis

Baseline characteristics were summarized using medians and interquartile ranges for continuous variables and n/N (%) for categorical variables. Between-group imbalance was evaluated using standardized mean differences (SMDs); for multi-level categorical variables, the maximum absolute category-level SMD was reported in the main baseline table as the variable-level SMD summary. Kaplan–Meier methods were used to describe time-to-event curves, and log-rank tests were used for unadjusted group comparisons in Kaplan–Meier analyses. Cox proportional hazards models were used for the primary endpoint analysis with facility-cluster robust standard errors using facility ID as the clustering variable. The model sequence included an unadjusted model, a model adjusted for the three-level genome medicine facility category, a clinical plus facility model adjusted for age, treatment start year, sex, ECOG PS, specimen type, pancreatic subtype group, and the three-level genome medicine facility category, and a sensitivity model additionally adjusted for log-transformed C-CAT pancreatic facility volume.

For the primary endpoint, a hazard ratio below 1 was interpreted as a lower hazard of recorded first-line treatment end, corresponding to longer recorded first-line treatment-process duration. Hazard ratios were interpreted only in relation to the operational endpoint definition and were not used to infer patient-level specialist involvement, treatment efficacy, survival benefit, facility quality ranking, or causality. Because the primary exposure was assigned at the facility level, facility-cluster robust standard errors were calculated using facility ID as the cluster variable. Within-facility correlation and residual facility-level confounding were considered important interpretive limitations. Sensitivity analyses included PAAD-only analyses, exclusion of other/non-adenocarcinoma cases while retaining missing/unspecified subtype cases, adjustment for log-transformed C-CAT pancreatic cohort volume per facility, complete recorded-end-date analysis, an extreme event-at-censor analysis, and alternative JSMO specialist-count thresholds. Median follow-up was estimated using the reverse Kaplan–Meier method in the supportive overall-survival cohort. Supportive OS was analyzed using the same facility-cluster robust Cox framework for contextual comparison. Cutoff sensitivity analyses evaluated alternative JSMO specialist-count thresholds. The primary comparison of 0–1 versus ≥2 specialists was prespecified as an operational threshold to distinguish facilities with limited specialist availability from those with at least a minimal multi-specialist presence and was not selected on the basis of *p* values. These sensitivity analyses were used to assess whether findings depended on the prespecified operational threshold and were not intended to identify an empirically optimized cutoff or to demonstrate a dose-response gradient. All *p* values were two-sided when calculated. Statistical analyses were performed using R version 4.5.2 (R Foundation for Statistical Computing, Vienna, Austria).

### 2.8. Ethical Considerations

This study used anonymized C-CAT data. Under the Japanese cancer genomic medicine system, patients provide informed consent before C-CAT registration. The study was approved by the Institutional Review Board of Toyama University on 1 May 2024 (Approval No. R2024016) and by the Information Utilization Review Board of C-CAT in August 2025 (Approval No. CDU2025-010N). Anonymized C-CAT data were accessed for research on 29 January 2026. The study was conducted in accordance with the Declaration of Helsinki, as revised in 2013. The authors did not have access to information that could identify individual participants during or after data collection.

## 3. Results

### 3.1. Study Cohort and Facility-Level Specialist Availability

**Figure 1:**
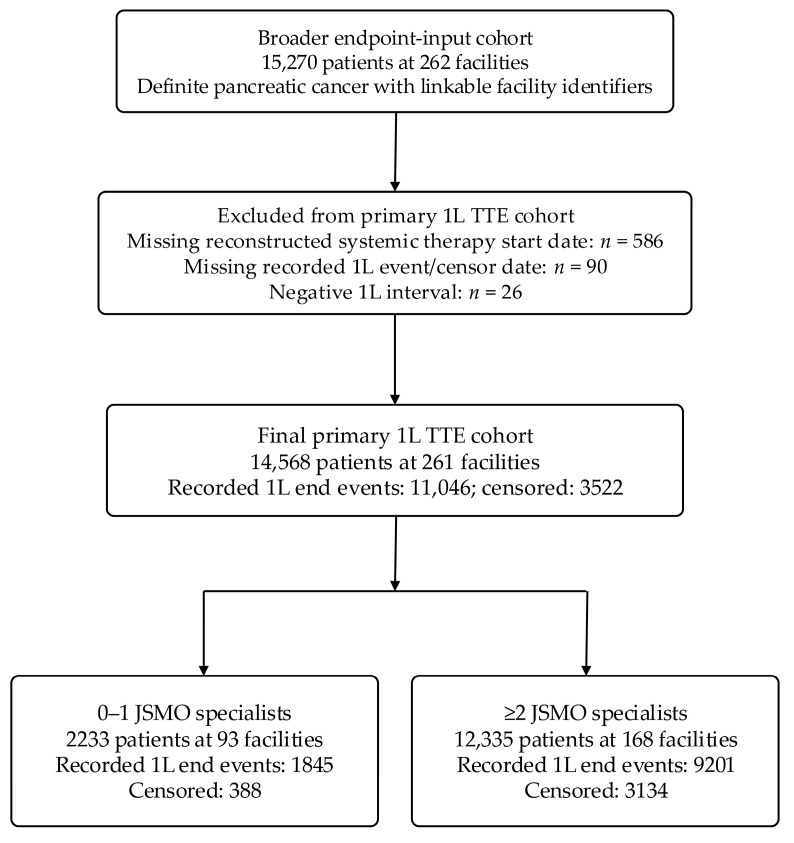
Patient-selection flowchart for the primary first-line time-to-event (1L TTE) analysis cohort. The broad endpoint-input cohort included 15,270 patients at 262 facilities with definite pancreatic cancer and linkable facility identifiers. Patients were excluded for missing reconstructed systemic therapy start date (*n* = 586), missing recorded 1L event/censor date after start-date exclusions (*n* = 90), or a negative 1L interval (*n* = 26), leaving 14,568 patients at 261 facilities in the primary analysis cohort.

The broader endpoint-input cohort comprised 15,270 patients at 262 facilities with definite pancreatic cancer and linkable facility identifiers. Patients were excluded from the primary 1L TTE cohort because of missing reconstructed systemic therapy start date (*n* = 586), missing recorded 1L event/censor date after start-date exclusions (*n* = 90), or a negative 1L interval (*n* = 26). The final primary first-line time-to-event analysis cohort included 14,568 patients at 261 facilities, with 11,046 recorded first-line treatment-end events and 3522 censored observations. The patient-selection flowchart is shown in Figure 1. In the primary cohort, 2233 patients were treated at 93 facilities with 0–1 registry-listed JSMO specialists, and 12,335 patients were treated at 168 facilities with ≥2 registry-listed JSMO specialists (Table 1). In the broader endpoint-input assessment, the corresponding groups included 2351 and 12,919 patients across 94 and 168 facilities, respectively. Endpoint denominator construction is summarized in Table 2 Panel A.

### 3.2. Baseline Characteristics

Baseline characteristics are summarized in Table 1, which now includes SMDs. The median age was 67 years overall and was similar between groups, with a slightly higher median age in the 0–1 specialist group than in the ≥2 specialist group (68 vs. 67 years). Male patients accounted for 55.4% of the overall cohort, with similar sex distributions across groups. The largest imbalances were observed for genome medicine facility category and the patient-level distribution of C-CAT pancreatic facility volume, with a smaller imbalance for ECOG PS. Facility-category distribution differed substantially by specialist availability: almost all patients in the 0–1 specialist group were treated at genome medicine cooperative hospitals, whereas the ≥2 specialist group included patients treated at cooperative, hub, and core hospitals. At the facility level, C-CAT pancreatic primary-cohort volume was higher in ≥2 specialist facilities than in 0–1 specialist facilities (median 42 vs. 17 patients per facility; Supplementary Table S5), although this measure represents C-CAT registry cohort volume rather than true institutional pancreatic cancer volume. Among ≥2 specialist facilities, the facility-level registry-listed JSMO specialist count had a mean of 4.9, median of 3, IQR of 2–6, and range of 2–34; patient-weighted values were higher (mean 8.6, median 7, IQR 3–10, range 2–34). FFPE specimens were more common in the ≥2 specialist group, while peripheral blood specimens were somewhat more common in the 0–1 specialist group. PAAD accounted for 12,118/14,568 patients (83.2%) in the primary cohort, missing/unspecified subtype for 1510 (10.4%) and other/non-adenocarcinoma subtype for 940 (6.5%); missing/unspecified subtype was not assumed to be PAAD.

### 3.3. Primary Endpoint: Time to Recorded First-Line Treatment End

**Figure 2:**
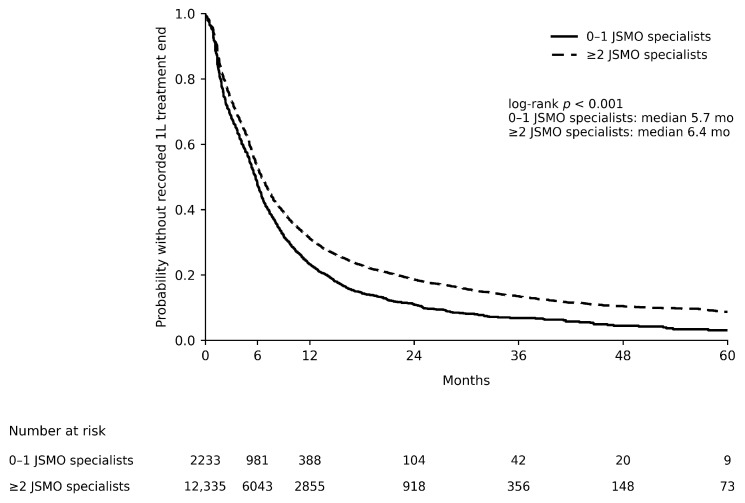
Kaplan–Meier curves for time from systemic therapy start to recorded first-line treatment end in the primary first-line time-to-event (1L TTE) analyzable cohort (*N* = 14,568). Curves are shown by facility-level registry-listed JSMO specialist availability, defined as 0–1 versus ≥2 JSMO specialists. The event was the recorded first-line treatment end; patients without a recorded 1L end-date were censored at death or last survival confirmation. The number-at-risk table shows patients with observed time ≥ t immediately before each displayed month. Median recorded first-line treatment-process duration was 5.7 months (95% CI 5.5–6.0) in the 0–1 specialist group and 6.4 months (95% CI 6.3–6.7) in the ≥2 specialist group; log-rank *p* < 0.001. JSMO, Japanese Society of Medical Oncology; 1L, first line.

Kaplan–Meier curves for time from systemic therapy start to recorded first-line treatment end are shown in Figure 2. The curve for the ≥2 specialist group showed a longer recorded first-line treatment-process duration than that for the 0–1 specialist group. Median recorded first-line treatment-process duration was 5.7 months (95% CI 5.5–6.0) in the 0–1 specialist group and 6.4 months (95% CI 6.3–6.7) in the ≥2 specialist group, with a log-rank *p* value < 0.001. The absolute median difference was 0.7 months. In Cox models using facility-cluster robust standard errors by facility ID, the association was present in the unadjusted model (HR 0.796, 95% CI 0.711–0.892; *p* < 0.001) and remained after adjustment for three-level genome medicine facility category (HR 0.899, 95% CI 0.814–0.993; *p* = 0.037) and after clinical plus facility adjustment (HR 0.895, 95% CI 0.810–0.988; *p* = 0.028) (Table 2). The clinical plus facility-adjusted association was statistically significant but modest in magnitude. The clinical plus facility plus log-volume sensitivity model was similar (HR 0.894, 95% CI 0.803–0.996; *p* = 0.042). Because the endpoint was recorded first-line treatment end, an HR <1 indicates a lower hazard of recorded first-line treatment end, corresponding to longer recorded first-line treatment-process duration. This finding should be interpreted as a facility-level association with an operational treatment-process endpoint and does not establish patient-level specialist involvement or causal effects.

In the PAAD-only sensitivity cohort (n = 12,118; 9207 events; 257 facilities), the clinical plus facility-adjusted Cox model with facility-cluster robust standard errors showed a directionally similar but attenuated association (HR 0.902, 95% CI 0.813–1.002; *p* = 0.054; Supplementary Table S2 and Supplementary Figure S1). Early Kaplan–Meier estimates showed a higher early recorded 1L end probability in 0–1 specialist facilities at 1–3 months, followed by later separation of the curves (Supplementary Table S7). Because PAAD accounted for most of the primary cohort, this attenuated result is clinically important and indicates that the primary all-pancreatic association was not clearly confirmed at conventional statistical significance within PAAD alone.

### 3.4. Cutoff Sensitivity Analysis

The 0–1 versus ≥2 cutoff was the prespecified operational definition of minimum plural specialist-team availability, intended to distinguish facilities with at least minimal plural specialist-team availability from those with no or only single listed specialist availability. Alternative cutoffs were examined as sensitivity analyses only and were not used to select the primary threshold. Using the clinical plus facility-adjusted Cox model with facility-cluster robust standard errors, alternative thresholds did not show a uniform monotonic pattern; the prespecified 0–1 versus ≥2 comparison was HR 0.895 (95% CI 0.810–0.988; *p* = 0.028), while other thresholds were close to the null, crossed 1.0, or varied in direction (Supplementary Table S1). Thus, the sensitivity analysis supports interpreting the ≥2-specialist cutoff as an a priori operational team-availability definition rather than as a data-optimized threshold or evidence of a linear dose-response relationship between specialist count and treatment-process duration.

### 3.5. Supportive Overall Survival Analysis

Supportive overall survival curves are shown in Supplementary Figure S2. The unadjusted log-rank *p* value for the supportive overall-survival curves was 0.011; this curve-level comparison is descriptive and was interpreted separately from the adjusted supportive OS model. The reverse Kaplan–Meier median follow-up in the supportive OS cohort was 32.3 months (95% CI 31.5–33.2). The median overall survival was 27.0 months in the 0–1 specialist group and 25.5 months in the ≥2 specialist group. The clinical plus facility-adjusted OS model with facility-cluster robust standard errors did not show a survival advantage for ≥2 specialist facilities (HR 1.056, 95% CI 0.933–1.194; *p* = 0.388; Supplementary Table S6). Thus, the adjusted supportive OS analysis did not support an interpretation that the longer recorded first-line treatment-process duration translated into a survival benefit.

### 3.6. Secondary/Exploratory Second-Line Treatment-Process Endpoint

The secondary/exploratory analysis of time from systemic therapy start to recorded second-line treatment end is summarized in Supplementary Table S8 and shown in Supplementary Figure S3. Median recorded second-line treatment-process duration was 18.4 months in the 0–1 specialist group and 21.1 months in the ≥2 specialist group, with a log-rank *p* value < 0.001. In the clinical plus facility-adjusted model with facility-cluster robust standard errors, the HR was 0.883 (95% CI 0.761–1.025; *p* = 0.102). This endpoint was included for comparability and hypothesis generation only. It reflects recorded treatment-process data and timing rather than treatment efficacy, survival benefit, or direct patient-level specialist involvement.

### 3.7. Endpoint Ascertainment and Denominator Audit

Endpoint ascertainment for the primary endpoint, including the denominator construction, is summarized in Table 2 Panel A. Of 15,270 patients in the broader endpoint-input cohort, 586 were excluded because the reconstructed systemic therapy start date was missing, 90 were excluded because the recorded first-line event or censoring date was missing, and 26 were excluded because of negative first-line intervals. The final primary first-line time-to-event denominator was therefore 14,568 patients at 261 facilities. Recorded 1L end-date availability differed between exposure groups: 1849/2351 patients (78.6%) in 0–1 specialist facilities and 9227/12,919 patients (71.4%) in ≥2 specialist facilities had a recorded 1L end-date. In the clinical plus facility-adjusted availability model among patients with reconstructed systemic therapy start dates, the OR for recorded 1L end-date availability in ≥2 versus 0–1 specialist facilities was 0.724 (95% CI 0.531–0.988; *p* = 0.042). Overall primary 1L TTE analyzability was similar (95.5% vs. 95.0%), and negative first-line intervals were rare. Endpoint sensitivity analyses were attenuated relative to the current primary analysis: the complete recorded-end-date sensitivity showed HR 0.982 (95% CI 0.890–1.083; *p* = 0.719), and the extreme event-at-censor sensitivity showed HR 0.928 (95% CI 0.858–1.004; *p* = 0.062) (Supplementary Table S3). These analyses indicate that endpoint ascertainment and the missing end-date mechanism should be considered when interpreting the primary recorded treatment-process endpoint.

## 4. Discussion

In this nationwide C-CAT pancreatic cancer facility-level analysis, availability of ≥2 registry-listed JSMO specialists was associated with a lower hazard of recorded first-line treatment end after clinical and facility adjustment with facility-cluster robust standard errors (HR, 0.895; 95% CI, 0.810–0.988; *p* = 0.028), corresponding to longer recorded first-line treatment-process duration. The absolute median difference was 0.7 months, and the adjusted association was statistically significant but modest in magnitude. Together, these results identify a measurable facility-level treatment-process signal associated with minimum plural JSMO specialist-team availability in a national cancer genomic medicine registry and demonstrate the feasibility of linking nationwide C-CAT real-world data with validated specialist-workforce variables to examine oncology care-delivery patterns in pancreatic cancer.

The primary endpoint was a recorded first-line treatment-process measure constructed from available C-CAT treatment records. In nationwide registry settings where progression-free survival and detailed treatment-failure reasons are not directly captured, time from systemic therapy start to recorded first-line treatment end can provide a pragmatic signal of care-delivery processes, including treatment-line documentation, clinical stability sufficient to continue or transition therapy, institutional workflows, referral pathways, and movement to subsequent treatment or supportive care. The endpoint therefore helps characterize how treatment-process data are recorded and differ across facility-level specialist-team contexts, provided its construction and censoring rules are reported transparently [7,12].

Registry-listed JSMO specialist availability provides a facility-level proxy for specialist-team infrastructure in systemic cancer therapy. Recent physician-level evidence from the SCRUM-Japan MONSTAR-SCREEN observational study reported that JSMO board certification of the enrolling physician was associated with longer overall survival in metastatic colorectal cancer despite comparable standard treatment implementation rates [13]. The present C-CAT analysis is complementary to that evidence because it examines a nationwide facility-level workforce signal within treatment-process data. The observed association is compatible with the interpretation that registry-listed plural specialist availability may serve as a marker of broader oncology care-delivery infrastructure, such as chemotherapy management capacity, multidisciplinary coordination, referral processes, and treatment documentation capacity.

Pancreatic cancer subtype heterogeneity is important when interpreting registry-defined pancreatic cancer cohorts. PAAD accounted for most patients in the primary cohort, but other/non-adenocarcinoma and missing/unspecified subtype cases were also present. The PAAD-only sensitivity analysis was directionally similar to the all-pancreatic analysis but attenuated. This pattern supports subtype-transparent presentation and sensitivity analysis and shows that the broad C-CAT pancreatic cohort and the clinically important PAAD subset provide complementary context.

Supportive overall survival provides contextual information for the primary treatment-process endpoint. The adjusted OS model did not show a corresponding survival advantage for facilities with ≥2 registry-listed JSMO specialists. This finding helps delineate the scope of the primary endpoint while preserving its value as a facility-level treatment-process or documentation-process signal. The secondary/exploratory second-line endpoint may provide hypothesis-generating context, but the main contribution of the study remains the facility-level association with the first-line record-derived treatment-process endpoint.

These findings highlight the utility of C-CAT for oncology care-delivery research. C-CAT can support nationwide facility-level analyses linking cancer genomic medicine data with validated facility-level workforce variables [9,10,11]. Such analyses are not a substitute for chemotherapy-specific clinical databases, but they can identify care-delivery signals that warrant direct evaluation in future data systems. Future national database elements should capture treatment regimen, treatment-line transition, dose modification, toxicity, discontinuation reason, progression, patient-level treating physician or team involvement, and facility-level care infrastructure, potentially informed by experience from national clinical database development [12,14].

This study has several strengths: a nationwide C-CAT dataset, a large pancreatic cancer cohort, linkage of patient-level registry data with validated facility-level JSMO specialist variables, a prespecified primary cutoff for minimum plural specialist-team availability, facility-cluster robust primary modeling, PAAD-only and endpoint-missingness sensitivity analyses, and transparent endpoint ascertainment and denominator construction.

### Limitations

Several limitations should be emphasized. First, the C-CAT cohort includes patients who underwent comprehensive genomic profiling and therefore is not the entire Japanese pancreatic cancer population [15,16,17]. CGP access and timing may be related to facility resources, clinical status, referral pathways, and survival long enough to undergo testing; selection into C-CAT may therefore differ by facility-level specialist availability and genome medicine infrastructure.

Second, the exposure was facility-level registry-listed JSMO specialist availability, not patient-level treating-physician certification or direct JSMO specialist involvement. Registry-listed specialist count does not capture all physicians who may treat pancreatic cancer, including non-JSMO-certified medical oncologists, and registry timing, affiliation status, or incomplete capture of affiliations may have affected counts.

Third, residual facility-level confounding remains substantial. The exposure groups differed strongly in genome medicine facility category and C-CAT pancreatic facility volume, and C-CAT pancreatic volume is not equivalent to true institutional pancreatic cancer volume. Genome medicine facility category is an administrative category of the cancer genomic medicine delivery system and should not be considered a direct measure of pancreatic cancer systemic-therapy capacity, multidisciplinary resources, facility quality, or pancreatic cancer volume. Facility-cluster robust standard errors address within-facility correlation in the Cox model but do not remove unmeasured confounding by hospital size, multidisciplinary resources, supportive care staffing, clinical trial access, referral patterns, chemotherapy management processes, or documentation capacity.

Fourth, the primary endpoint was recorded first-line treatment-process duration and should not be interpreted as progression-free survival, treatment efficacy, survival, or quality of care. The modest endpoint difference cannot determine whether the observed pattern reflects treatment management, documentation practice, or patient benefit. Reasons for recorded first-line treatment end and the missing end-date mechanism were unavailable; recorded end-date availability differed by exposure group, and complete recorded-end-date and extreme event-at-censor sensitivity analyses attenuated the association. Supportive overall survival did not show a corresponding adjusted survival advantage. These issues limit patient-benefit claims and support interpreting the endpoint as a record-derived process measure.

Fifth, during construction of the final analysis dataset, treatment regimen, FOLFIRINOX, gemcitabine/nab-paclitaxel, dose intensity, toxicity, radiologic progression, discontinuation reason, resection status, and metastatic burden could not be reliably derived as structured analysis variables. Stage before first treatment had substantial missing/unknownness and was not used in the primary adjustment. PAAD was the dominant subtype but not exclusive, and the PAAD-only sensitivity analysis was attenuated; therefore, PAAD-specific inference remains limited.

Finally, as an observational registry-based facility-level analysis, this study cannot establish causality, evaluate facility quality, or determine the effect of increasing JSMO specialist availability on treatment outcomes. The findings should be interpreted as hypothesis-generating facility-level associations in record-derived treatment-process data.

## 5. Conclusions

In this nationwide C-CAT registry-based facility-level analysis of pancreatic cancer, availability of ≥2 registry-listed JSMO specialists was associated with a lower hazard of recorded first-line treatment end after clinical and facility adjustment with facility-cluster robust standard errors, corresponding to longer recorded first-line treatment-process duration. These findings suggest that facility-level availability of multiple JSMO specialists may reflect broader oncology-care infrastructure associated with treatment-process characteristics. Confirmation of any patient-level clinical benefit will require dedicated prospective datasets including detailed treatment and physician-level information.

## Figures and Tables

**Table 1:** Baseline characteristics of the primary first-line time-to-event (1L TTE) cohort by facility-level minimum JSMO specialist-team availability, including standardized mean differences.

Variable	Level	0–1 JSMO Specialists	≥2 JSMO Specialists	SMD
Patients		2233	12,335	
Facilities		93	168	
Age, years	Median (IQR)	68.0 (60.0–74.0)	67.0 (59.0–73.0)	−0.068
Treatment start year	Median (IQR)	2022 (2021–2023)	2022 (2020–2023)	−0.149
C-CAT pancreatic facility volume (primary 1L cohort)	Median (IQR)	47 (21–61)	114 (67–234)	1.114
Sex	Man	1277/2233 (57.2%)	6800/12,335 (55.1%)	0.042
Sex	Woman	956/2233 (42.8%)	5534/12,335 (44.9%)	
Sex	Missing/unknown	0/2233 (0.0%)	1/12,335 (0.0%)	
ECOG PS category	0	1547/2233 (69.3%)	7087/12,335 (57.5%)	0.245
ECOG PS category	1	604/2233 (27.0%)	4750/12,335 (38.5%)	
ECOG PS category	2	41/2233 (1.8%)	300/12,335 (2.4%)	
ECOG PS category	≥3	12/2233 (0.5%)	40/12,335 (0.3%)	
ECOG PS category	Missing/unknown	29/2233 (1.3%)	158/12,335 (1.3%)	
Genome medicine facility category	Genome medicine cooperative hospital	2117/2233 (94.8%)	5015/12,335 (40.7%)	1.158
Genome medicine facility category	Genome medicine hub hospital	116/2233 (5.2%)	4493/12,335 (36.4%)	
Genome medicine facility category	Genome medicine core hospital	0/2233 (0.0%)	2827/12,335 (22.9%)	
Specimen type	FFPE	1485/2233 (66.5%)	8851/12,335 (71.8%)	0.114
Specimen type	Peripheral blood	745/2233 (33.4%)	3467/12,335 (28.1%)	
Specimen type	Fresh frozen	2/2233 (0.1%)	12/12,335 (0.1%)	
Specimen type	Other	1/2233 (0.0%)	5/12,335 (0.0%)	
Pancreatic subtype group	PAAD	1947/2233 (87.2%)	10,171/12,335 (82.5%)	0.168
Pancreatic subtype group	Missing/unspecified subtype	142/2233 (6.4%)	1368/12,335 (11.1%)	
Pancreatic subtype group	Other/non-adenocarcinoma	144/2233 (6.4%)	796/12,335 (6.5%)	

Values are shown as n/N (%) unless otherwise indicated. Continuous variables are shown as median (IQR). SMD denotes standardized mean difference; for multi-level categorical variables, the maximum absolute category-level SMD is shown in the main table. Minimum specialist-team availability was defined as facility-level registry-listed JSMO specialist count of 0–1 versus ≥2 specialists. ECOG PS 2, 3, and 4 were collapsed into ≥2. Fresh frozen and other specimen types were collapsed into Other. Nonblank non-PAAD pancreatic subtypes were collapsed into Other/non-adenocarcinoma. The C-CAT pancreatic facility-volume row describes the patient-level distribution of the volume of the facility to which each patient belonged; facility-level summaries are provided in Supplementary Table S5. Genome medicine facility categories were grouped as core hospitals, hub hospitals, and cooperative hospitals. The cooperative hospital category was treated as a broad cooperative/liaison category within the Japanese cancer genomic medicine delivery system, and subcategories within cooperative hospitals were not modeled separately. This facility-category variable was used for broad facility-level adjustment and should not be interpreted as a facility-quality ranking or as a direct measure of pancreatic cancer treatment capability. Minimum specialist-team availability is a facility-level registry-listed proxy and does not establish patient-level specialist involvement. 1L, first-line; C-CAT, Center for Cancer Genomics and Advanced Therapeutics; ECOG PS, Eastern Cooperative Oncology Group performance status; FFPE, formalin-fixed paraffin-embedded; IQR, interquartile range; JSMO, Japanese Society of Medical Oncology; PAAD, pancreatic adenocarcinoma; SMD, standardized mean difference; TTE, time to event.

**Table 2:** Primary endpoint ascertainment and facility-cluster robust Cox models for time from systemic therapy start to recorded first-line treatment end.

**Panel A. Primary Endpoint Ascertainment Only.**					
**Metric**	**0–1 JSMO Specialists**	**≥2 JSMO Specialists**	**Interpretive Note**		
Broader endpoint-input cohort cases	2351	12,919	Denominator for endpoint-input assessment.		
Facilities	94	168	Unique C-CAT facility IDs.		
Systemic therapy start date available	2242/2351 (95.4%)	12,442/12,919 (96.3%)	Start date reconstructed from treatment records.		
Recorded 1L end-date available	1849/2351 (78.6%)	9227/12,919 (71.4%)	Primary event-date availability; missing does not mean ongoing 1L therapy.		
Event/censor date available for 1L endpoint	2335/2351 (99.3%)	12,780/12,919 (98.9%)	Recorded 1L end-date or death/last survival confirmation date.		
Primary 1L TTE analyzable	2233/2351 (95.0%)	12,335/12,919 (95.5%)	Systemic therapy start, 1L event/censor date, and non-negative interval.		
Primary 1L events among analyzable	1845/2233 (82.6%)	9201/12,335 (74.6%)	Recorded 1L end events.		
Primary 1L censored among analyzable	388/2233 (17.4%)	3134/12,335 (25.4%)	Censored at death or last survival confirmation when 1L end-date was unavailable.		
Negative 1L intervals flagged and excluded	4/2351 (0.2%)	22/12,919 (0.2%)	Flagged and excluded from primary denominator.		
**Panel B. Primary facility-cluster robust Cox models.**					
**Model**	**N**	**Events**	**Facilities**	**HR (95% CI)**	***p* value**
Unadjusted	14,568	11,046	261	0.796 (0.711–0.892)	<0.001
Facility adj.	14,568	11,046	261	0.899 (0.814–0.993)	0.037
Clinical + facility adj.	14,568	11,046	261	0.895 (0.810–0.988)	0.028
Clinical + facility + volume adj.	14,568	11,046	261	0.894 (0.803–0.996)	0.042

The broader endpoint-input cohort was used for endpoint-input assessment. Recorded 1L end-date absence should not be interpreted as ongoing first-line therapy. Metrics are descriptive endpoint-ascertainment/data-system measures rather than comparative assessments of facility quality. JSMO, Japanese Society of Medical Oncology; TTE, time to event; 1L, first-line treatment. HR <1 indicates a lower hazard of recorded first-line treatment end, corresponding to longer recorded first-line treatment-process duration. All Cox models used facility-cluster robust standard errors by facility ID. The facility-category model was adjusted for three-level genome medicine facility category. The clinical plus facility model was adjusted for age, treatment start year, sex, ECOG PS, specimen type, pancreatic subtype group, and three-level genome medicine facility category. The log(volume) sensitivity model additionally adjusted for log-transformed C-CAT pancreatic facility volume. This is not PFS, treatment efficacy, OS benefit, causality, facility quality ranking, or patient-level specialist involvement. CI, confidence interval; ECOG PS, Eastern Cooperative Oncology Group performance status; HR, hazard ratio; JSMO, Japanese Society of Medical Oncology.

## Data Availability

The authors do not control the underlying C-CAT database. The anonymized C-CAT data analyzed in this study were obtained through the approved C-CAT data-use framework and are subject to institutional and data-provider governance restrictions. Accordingly, the source-level data are not publicly available from the authors. Access to C-CAT data requires a formal application to C-CAT and approval by the relevant institutional and data-use review bodies under the Japanese cancer genomic medicine governance framework.

## Supplementary Materials

The following supporting information can be downloaded at: https://www.mdpi.com/article/10.3390/curroncol33070393/s1, Supplementary Table S1: Cutoff sensitivity analysis for the primary first-line time-to-event (1L TTE) endpoint; Supplementary Table S2: PAAD-only and subtype-restricted sensitivity analysis; Supplementary Table S3: Endpoint ascertainment and missing end-date sensitivity analyses; Supplementary Table S4: Missing covariate handling and unavailable treatment-variable audit; Supplementary Table S5: Facility volume and JSMO specialist-count distributions; Supplementary Table S6: Follow-up and supportive overall survival model; Supplementary Table S7: Early KM estimates and curve-crossing context; Supplementary Table S8: Secondary/exploratory second-line time-to-event (2L TTE) summary; Supplementary Figure S1: PAAD-only primary first-line time-to-event (1L TTE) Kaplan–Meier curve; Supplementary Figure S2: Supportive overall survival Kaplan–Meier curve; Supplementary Figure S3: Secondary/exploratory 2L TTE Kaplan–Meier curve.
