# Supplementary material for: Facility-Level Availability of Japanese Society of Medical Oncology Specialists and Recorded First-Line Treatment-Process Duration in Pancreatic Cancer: A Nationwide Center for Cancer Genomics and Advanced Therapeutics Registry Analysis
Source: Curr Oncol. 2026 Jul 1;33(7):393. doi: 10.3390/curroncol33070393 (PMC13409419; doi:10.3390/curroncol33070393)
Supplement: Supplementary file 1 [file curroncol-33-00393-s001.zip › 37_CO_supplementary_materials_20260615e.pdf]

Supplementary Materials

Facility-Level Availability of Japanese Society of Medical Oncology Specialists and Recorded First-Line Treatment-Process Duration in Pancreatic Cancer: A Nationwide Center for Cancer Genomics and Advanced Therapeutics Registry Analysis

List of supplementary tables and figures

Supplementary Table S1. Cutoff sensitivity analysis for the primary first-line time-to-event (1L TTE) endpoint.

Supplementary Table S2. PAAD-only and subtype-restricted sensitivity analysis.

Supplementary Table S3. Endpoint ascertainment and missing end-date sensitivity analyses.

Supplementary Table S4. Missing covariate handling and unavailable treatment-variable audit.

Supplementary Table S5. Facility volume and JSMO specialist-count distributions.

Supplementary Table S6. Follow-up and supportive overall survival model.

Supplementary Table S7. Early KM estimates and curve-crossing context.

Supplementary Table S8. Secondary/exploratory second-line time-to-event (2L TTE) summary.

Supplementary Figure S1. PAAD-only primary first-line time-to-event (1L TTE) Kaplan–Meier curve.

Supplementary Figure S2. Supportive overall survival Kaplan–Meier curve.

Supplementary Figure S3. Secondary/exploratory 2L TTE Kaplan–Meier curve.

Supplementary figures are submitted separately as TIFF files; legends are provided below.

Supplementary Table S1. Cutoff sensitivity analysis for the primary first-line time-to-event (1L TTE) endpoint.

Cutoff sensitivity

Cutoff definition	Role	Low group patients	High group patients	Low group facilities	High group facilities	Low group events	High group events	Clinical + facility adjusted HR (95% CI)	p value	Robust SE note
0 vs ≥1	Sensitivity only	596	13,972	32	229	480	10,566	0.985 (0.863–1.125)	p = 0.824	Facility-cluster robust SE by facility.
0–1 vs ≥2	Primary exposure	2,233	12,335	93	168	1,845	9,201	0.895 (0.810–0.988)	p = 0.028	Facility-cluster robust SE by facility.
0–2 vs ≥3	Sensitivity only	4,143	10,425	147	114	3,252	7,794	1.033 (0.925–1.154)	p = 0.565	Facility-cluster robust SE by facility.

Cutoff definition	Role	Low group patients	High group patients	Low group facilities	High group facilities	Low group events	High group events	Clinical + facility adjusted HR (95% CI)	p value	Robust SE note
0–3 vs ≥4	Sensitivity only	5,658	8,910	181	80	4,488	6,558	0.980 (0.869–1.105)	p = 0.743	Facility-cluster robust SE by facility.
0–4 vs ≥5	Sensitivity only	6,850	7,718	203	58	5,434	5,612	0.981 (0.854–1.126)	p = 0.782	Facility-cluster robust SE by facility.
0–5 vs ≥6	Sensitivity only	7,490	7,078	213	48	5,954	5,092	0.943 (0.803–1.108)	p = 0.475	Facility-cluster robust SE by facility.
0–6 vs ≥7	Sensitivity only	7,996	6,572	223	38	6,382	4,664	0.894 (0.753–1.062)	p = 0.203	Facility-cluster robust SE by facility.
0–7 vs ≥8	Sensitivity only	9,183	5,385	233	28	7,211	3,835	0.961 (0.798–1.158)	p = 0.676	Facility-cluster robust SE by facility.

Note: The 0–1 versus ≥2 cutoff was selected a priori as an operational definition of minimum plural specialist-team availability. Alternative thresholds are exploratory sensitivity checks only. HR <1 indicates a lower hazard of recorded first-line treatment end.

Supplementary Table S2. PAAD-only and subtype-restricted sensitivity analysis.

Sensitivity cohorts and adjusted Cox model results

Cohort definition	Patients	Facilities	Events	Censored	0–1 patients	≥2 patients	PAAD n	Missing/unspecified subtype n	Other/non-adenocarcinoma n	Clinical + facility adjusted HR (95% CI)	p value
All pancreatic cohort	14,568	261	11,046	3,522	2,233	12,335	12,118	1,510	940	0.895 (0.810–0.988)	p = 0.028
PAAD-only cohort	12,118	257	9,207	2,911	1,947	10,171	12,118	0	0	0.902 (0.813–1.002)	p = 0.054
Exclude other/non-adenocarcinoma; retain missing/unspecified subtype	13,628	258	10,389	3,239	2,089	11,539	12,118	1,510	0	0.895 (0.809–0.990)	p = 0.031

Note: Missing/unspecified pancreatic subtype was not assumed to be PAAD. HR <1 indicates a lower hazard of recorded first-line treatment end. Models used clinical plus facility adjustment with facility-cluster robust standard errors.

Supplementary Table S3. Endpoint ascertainment and missing end-date sensitivity analyses.

Panel A. Key endpoint ascertainment values

Endpoint ascertainment metric	Overall	0–1 JSMO specialists	≥2 JSMO specialists	Interpretive note
Broad endpoint-input cohort	15,270/15,270 (100.0%)	2,351/2,351 (100.0%)	12,919/12,919 (100.0%)	Reference denominator.
Systemic therapy start date available	14,684/15,270 (96.2%)	2,242/2,351 (95.4%)	12,442/12,919 (96.3%)	Input required for endpoint construction.
Recorded 1L end date available	11,076/15,270 (72.5%)	1,849/2,351 (78.6%)	9,227/12,919 (71.4%)	Recorded first-line end-date availability differed by exposure group.
Event/censor date available for 1L endpoint	15,115/15,270 (99.0%)	2,335/2,351 (99.3%)	12,780/12,919 (98.9%)	Death or last survival confirmation allowed censoring when no 1L end date was recorded.
Primary 1L TTE analyzable	14,568/15,270 (95.4%)	2,233/2,351 (95.0%)	12,335/12,919 (95.5%)	Final primary endpoint analyzability.
Primary 1L events	11,046/14,568 (75.8%)	1,845/2,233 (82.6%)	9,201/12,335 (74.6%)	Recorded first-line treatment-end events in the primary cohort.
Primary 1L censored	3,522/14,568 (24.2%)	388/2,233 (17.4%)	3,134/12,335 (25.4%)	Censored observations in the primary cohort.
Negative 1L interval	26/15,270 (0.2%)	4/2,351 (0.2%)	22/12,919 (0.2%)	Excluded from primary endpoint construction.
Among without recorded 1L end date: death date available	1,771/4,194 (42.2%)	197/502 (39.2%)	1,574/3,692 (42.6%)	Among patients without a recorded 1L end date.
Among without recorded 1L end date: last survival confirmation date available	2,872/4,194 (68.5%)	344/502 (68.5%)	2,528/3,692 (68.5%)	Among patients without a recorded 1L end date.
Among without recorded 1L end date: insufficient endpoint/censor date	155/4,194 (3.7%)	16/502 (3.2%)	139/3,692 (3.8%)	Among patients without a recorded 1L end date.

Panel B. Endpoint-missingness sensitivity Cox analyses

Analysis	Cohort	N	Events	Facilities	Clinical + facility adjusted HR (95% CI)	p value	Note
Current primary analysis	All-pancreatic primary 1L TTE cohort	14,568	11,046	261	0.895 (0.810–0.988)	p = 0.028	Facility-cluster robust SE by facility.
Complete recorded-end-date sensitivity	Patients with recorded 1L end date	11,046	11,046	260	0.982 (0.890–1.083)	p = 0.719	Facility-cluster robust SE by facility.
Extreme event-at-censor sensitivity	Primary cohort, censored observations treated as events	14,568	14,568	261	0.928 (0.858–1.004)	p = 0.062	Facility-cluster robust SE by facility.

Panel C. Recorded 1L end-date availability models

Model	Denominator	N	Recorded 1L end-date events	Facilities	OR for recorded 1L end-date availability (≥2 vs 0–1) (95% CI)	p value	Note
Unadjusted	Broad cohort with systemic therapy start date	14,684	11,068	261	0.608 (0.448–0.825)	p = 0.001	Cluster-robust SE by facility.
Clinical + facility adjusted	Broad cohort with systemic therapy start date	14,684	11,068	261	0.724 (0.531–0.988)	p = 0.042	Cluster-robust SE by facility.
Clinical + facility + log(volume)	Broad cohort with systemic therapy start date	14,684	11,068	261	0.732 (0.530–1.011)	p = 0.059	Cluster-robust SE by facility.

Note: Missing recorded 1L end-date mechanism could not be directly determined from the available C-CAT fields. Sensitivity analyses are interpreted as endpoint-construct validity checks, not as new causal analyses.

Supplementary Table S4. Missing covariate handling and unavailable treatment-variable audit.

Panel A. Missing covariate handling

Variable	Column used	Missing/unknown n	Denominator	Percent missing	Handling in analysis
age	age	0	14,568	0.0%	Continuous; complete cases used.
treatment start year	treatment_start_year	0	14,568	0.0%	Continuous; complete cases used.
sex	sex_category	1	14,568	0.0%	Categorical; missing/unknown retained as a category.
ECOG PS	ecog_ps_category	187	14,568	1.3%	Categorical; missing/unknown retained as a category.
pancreatic subtype group	subtype_group	1,510	14,568	10.4%	Categorical; missing/unspecified retained as a category in all-pancreatic models; omitted in PAAD-only model.
specimen type	specimen_type_category	0	14,568	0.0%	Categorical; rare categories retained in descriptive table; combined as other/fresh frozen in adjusted models.
stage before first treatment	stage_before_first_treatment	9,837	14,568	67.5%	Not included in main models because of high missing/unknownness.
genome medicine facility category	facility_category_3level	0	14,568	0.0%	Categorical; complete in primary cohort.
facility ID	facility_id	0	14,568	0.0%	Cluster variable; complete in primary cohort.
log C-CAT pancreatic facility volume	log_facility_primary_1L_volume	0	14,568	0.0%	Continuous sensitivity covariate; derived from primary 1L TTE cohort volume.

Panel B. Unavailable treatment-variable audit

Requested variable	Availability	Available column if any	Action taken
regimen name	Not available in the C-CAT analysis dataset	None identified	No reliable structured variable found; no parsing attempted.
drug names	Not available in the C-CAT analysis dataset	None identified	No reliable structured variable found; no parsing attempted.
FOLFIRINOX	Not available in the C-CAT analysis dataset	None identified	No reliable structured variable found; no parsing attempted.
gemcitabine	Not available in the C-CAT analysis dataset	None identified	No reliable structured variable found; no parsing attempted.
nab-paclitaxel	Not available in the C-CAT analysis dataset	None identified	No reliable structured variable found; no parsing attempted.
dose intensity	Not available in the C-CAT analysis dataset	None identified	No reliable structured variable found; no parsing attempted.
toxicity	Not available in the C-CAT analysis dataset	None identified	No reliable structured variable found; no parsing attempted.

Requested variable	Availability	Available column if any	Action taken
progression	Not available in the C-CAT analysis dataset	None identified	No reliable structured variable found; no parsing attempted.
discontinuation reason	Not available in the C-CAT analysis dataset	None identified	No reliable structured variable found; no parsing attempted.
resection status	Not available in the C-CAT analysis dataset	None identified	No reliable structured variable found; no parsing attempted.
metastatic burden	Only stage_before_first_treatment field found; not a reliable metastatic-burden variable for main models because missing/unknownness is high.	stage_before_first_treatment	Stage before first treatment has 67.5% missing/unknown in the primary cohort.

Note: Missing/unknown categories were retained for categorical covariates when used in the main all-pancreatic models. Multiple imputation was not performed because missingness included structural unknown/unspecified categories and the analysis objective was descriptive sensitivity assessment rather than new model development.

Supplementary Table S5. Facility volume and JSMO specialist-count distributions.

Panel A. Facility-level C-CAT pancreatic cohort volume

Cohort	Exposure group	Facilities	Patients	Mean facility C-CAT pancreatic volume (SD)	Median facility volume (IQR)	Min–Max
Broad endpoint-input cohort	Overall	262	15,270	58.3 (80.8)	29.0 (16.0–72.0)	1–672
Broad endpoint-input cohort	0–1 JSMO specialists	94	2,351	25.0 (26.8)	18.5 (7.0–27.0)	1–132
Broad endpoint-input cohort	≥2 JSMO specialists	168	12,919	76.9 (94.0)	44.0 (24.0–99.2)	2–672
Primary 1L TTE cohort	Overall	261	14,568	55.8 (77.0)	29.0 (16.0–66.0)	1–662
Primary 1L TTE cohort	0–1 JSMO specialists	93	2,233	24.0 (24.1)	17.0 (7.0–27.0)	1–116
Primary 1L TTE cohort	≥2 JSMO specialists	168	12,335	73.4 (89.6)	42.0 (22.8–97.2)	2–662

Panel B. Facility-level and patient-weighted JSMO specialist-count distributions

Weighting	Exposure group	N facilities or patients	Mean JSMO specialist count (SD)	Median specialist count (IQR)	Min–Max
Facility-level	Overall	261	3.4 (4.2)	2.0 (1.0–4.0)	0–34
Facility-level	0–1 JSMO specialists	93	0.7 (0.5)	1.0 (0.0–1.0)	0–1
Facility-level	≥2 JSMO specialists	168	4.9 (4.6)	3.0 (2.0–6.0)	2–34
Patient-weighted	Overall	14,568	7.4 (7.6)	5.0 (2.0–10.0)	0–34
Patient-weighted	0–1 JSMO specialists	2,233	0.7 (0.4)	1.0 (0.0–1.0)	0–1
Patient-weighted	≥2 JSMO specialists	12,335	8.6 (7.7)	7.0 (3.0–10.0)	2–34

Note: C-CAT pancreatic cohort volume is a registry cohort measure and should not be interpreted as true institutional pancreatic cancer volume. Patient-weighted specialist-count values describe the distribution among patients, not facilities.

Supplementary Table S6. Follow-up and supportive overall survival model.

Panel A. Reverse Kaplan–Meier follow-up

Group	N	Deaths	Censored	Facilities	Reverse KM median follow-up, months (95% CI)	Follow-up probability at 6 months	12 months	24 months
Overall	14,367	7,967	6,400	258	32.3 (31.5–33.2)	95.5%	85.1%	62.2%

Group	N	Deaths	Censored	Facilities	Reverse KM median follow-up, months (95% CI)	Follow-up probability at 6 months	12 months	24 months
0–1 JSMO specialists	2,180	1,156	1,024	92	32.2 (30.1–33.7)	95.4%	84.2%	62.1%
≥2 JSMO specialists	12,187	6,811	5,376	166	32.4 (31.5–33.4)	95.5%	85.3%	62.2%

Panel B. Supportive OS Kaplan–Meier summary and adjusted Cox model

Component	Group/model	N	Events	Censored	Facilities	Median OS months (95% CI)	Effect estimate	p value	Note
KM median OS	0–1 JSMO specialists	2,180	1,156	1,024	92	27.0 (25.4–28.7)		log-rank p = 0.011	Supportive curve-level comparison only.
KM median OS	≥2 JSMO specialists	12,187	6,811	5,376	166	25.5 (25.0–26.1)			Supportive curve-level comparison only.
Adjusted Cox model	≥2 versus 0–1 JSMO specialists	14,365	7,967		258		1.056 (0.933–1.194)	p = 0.388	Facility-cluster robust SE by facility; supportive context only.

Note: Supportive OS was not used to infer a survival advantage. The adjusted supportive OS model did not show a survival advantage for ≥2 specialist facilities.

Supplementary Table S7. Early KM estimates and curve-crossing context.

Early primary 1L TTE Kaplan–Meier estimates

Group	Time point, months	Number at risk	Cumulative recorded 1L end events	KM probability without recorded 1L end	Cumulative recorded 1L end probability
0–1 JSMO specialists	1	2,048	183	91.8%	8.2%
0–1 JSMO specialists	2	1,714	514	77.0%	23.0%
0–1 JSMO specialists	3	1,498	714	67.9%	32.1%
0–1 JSMO specialists	6	981	1,152	47.5%	52.5%
0–1 JSMO specialists	12	388	1,617	23.2%	76.8%
≥2 JSMO specialists	1	11,555	773	93.7%	6.3%
≥2 JSMO specialists	2	9,974	2,303	81.3%	18.7%
≥2 JSMO specialists	3	8,874	3,296	73.2%	26.8%
≥2 JSMO specialists	6	6,043	5,701	52.8%	47.2%
≥2 JSMO specialists	12	2,855	7,998	31.1%	68.9%

Note: These estimates describe early curve behavior only. No mechanism is inferred from early curve crossing/convergence.

Supplementary Table S8. Secondary/exploratory 2L TTE summary.

Secondary/exploratory 2L TTE Kaplan–Meier and Cox summary

Component	Group/model	N	Events	Censored	Facilities	Median months (95% CI)	Effect estimate	p value	Note
KM median recorded 2L TTE	0–1 JSMO specialists	2,215	1,158	1,057	93	18.4 (16.9–19.7)		log-rank p < 0.001	Secondary/exploratory endpoint.
KM median recorded 2L TTE	≥2 JSMO specialists	12,257	5,679	6,578	168	21.1 (20.5–21.8)			Secondary/exploratory endpoint.
Adjusted Cox model	≥2 versus 0–1 JSMO specialists	14,472	6,837	7,635	261		0.883 (0.761–1.025)	p = 0.102	Facility-cluster robust SE by facility; secondary/exploratory endpoint.

Note: This endpoint is secondary/exploratory and is included for comparability and hypothesis generation only. It reflects recorded treatment-process data rather than treatment efficacy or survival benefit.

Supplementary Figure Legends

Supplementary Figure S1. Kaplan–Meier curves for time from systemic therapy start to recorded first-line treatment end in the PAAD-only subset of the primary 1L TTE analyzable cohort (N=12,118). Missing/unspecified pancreatic subtype was not included as PAAD. Curves are shown by facility-level registry-listed JSMO specialist availability, defined as 0–1 versus ≥2 JSMO specialists. The event was recorded first-line treatment end; patients without a recorded 1L end date were censored at death or last survival confirmation. The number-at-risk table shows patients with observed time ≥ t immediately before each displayed month. Median recorded first-line treatment-process duration was 5.8 months (95% CI 5.5–6.0) in the 0–1 specialist group and 6.4 months (95% CI 6.2–6.7) in the ≥2 specialist group; log-rank p < 0.001. PAAD, pancreatic adenocarcinoma; JSMO, Japanese Society of Medical Oncology; TTE, time to event; 1L, first line; CI, confidence interval.

Supplementary Figure S2. Kaplan–Meier curves for supportive overall survival from systemic therapy start in the OS-calculable cohort (N=14,367). Curves are shown by facility-level registry-listed JSMO specialist availability, defined as 0–1 versus ≥2 JSMO specialists. The event was death; patients without a recorded death date were censored at the last survival confirmation date. The number-at-risk table shows patients with observed time ≥ t immediately before each displayed month. Median OS was 27.0 months (95% CI 25.4–28.7) in the 0–1 specialist group and 25.5 months (95% CI 25.0–26.1) in the ≥2 specialist group; unadjusted log-rank p = 0.011. This supportive curve-level comparison was not used as evidence of a survival advantage and should be interpreted alongside the adjusted supportive OS model. OS, overall survival; JSMO, Japanese Society of Medical Oncology; CI, confidence interval.

Supplementary Figure S3. Kaplan–Meier curves for time from systemic therapy start to recorded second-line treatment end in the secondary/exploratory 2L TTE analyzable cohort (N=14,472). Curves are shown by facility-level registry-listed JSMO specialist availability, defined as 0–1 versus ≥2 JSMO specialists. The event was recorded second-line treatment end; patients without a recorded 2L end date were censored at death or last survival confirmation. The number-at-risk table shows patients with observed time ≥ t immediately before each displayed month. Median recorded second-line treatment-process duration was 18.4 months (95% CI 16.9–19.7) in the 0–1 specialist group and 21.1 months (95% CI 20.5–21.8) in the ≥2 specialist group; log-rank p < 0.001. JSMO, Japanese Society of Medical Oncology; TTE, time to event; 2L, second line; CI, confidence interval.
