# Supplementary figures and images for: Facility-Level Availability of Japanese Society of Medical Oncology Specialists and Recorded First-Line Treatment-Process Duration in Pancreatic Cancer: A Nationwide Center for Cancer Genomics and Advanced Therapeutics Registry Analysis
Source: Curr Oncol. 2026 Jul 1;33(7):393. doi: 10.3390/curroncol33070393 (PMC13409419; doi:10.3390/curroncol33070393)

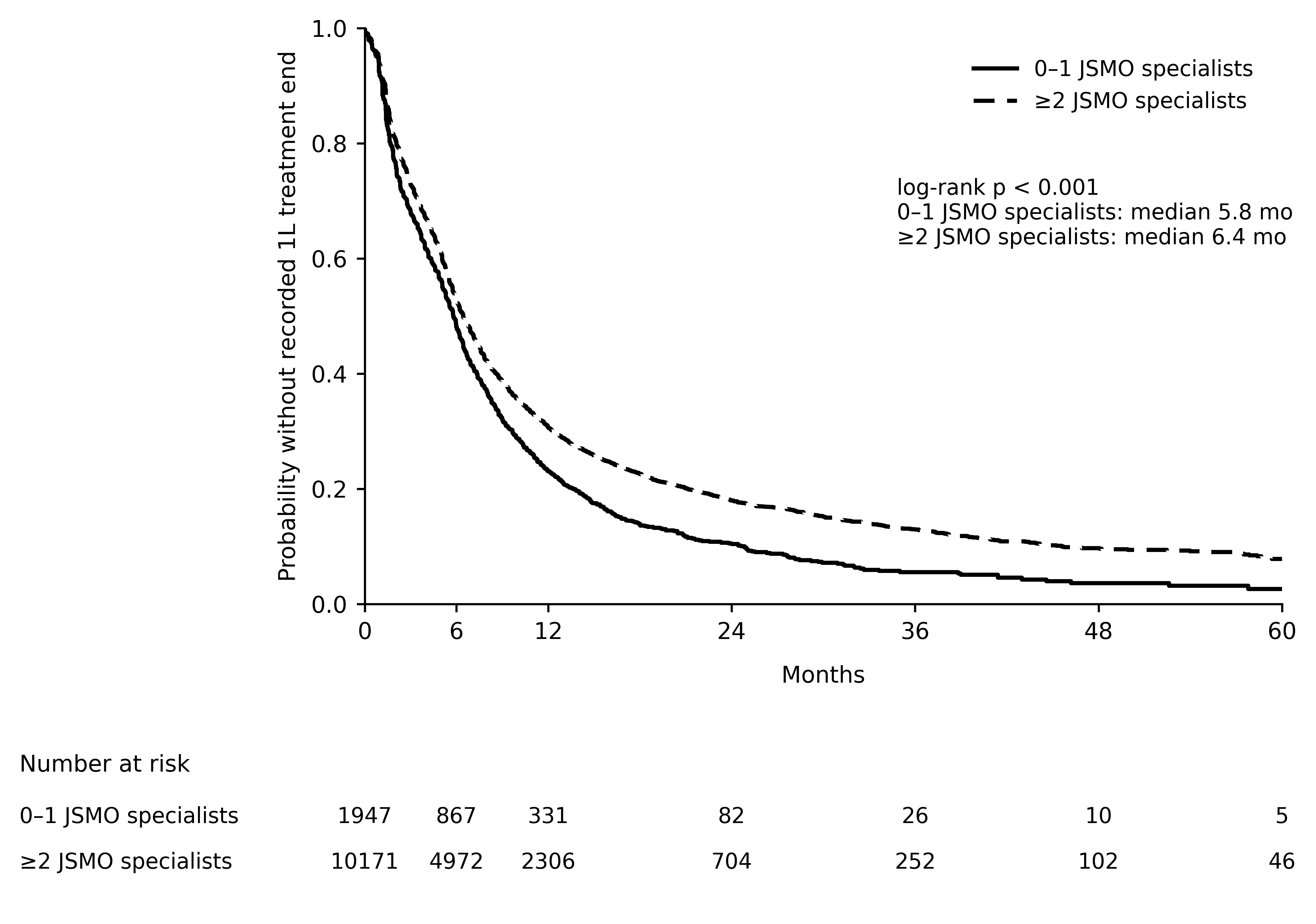

Supplement: Supplementary file 1 [file curroncol-33-00393-s001.zip › 37_CO_SFigure S1_PAAD_1L_20260614.tiff]

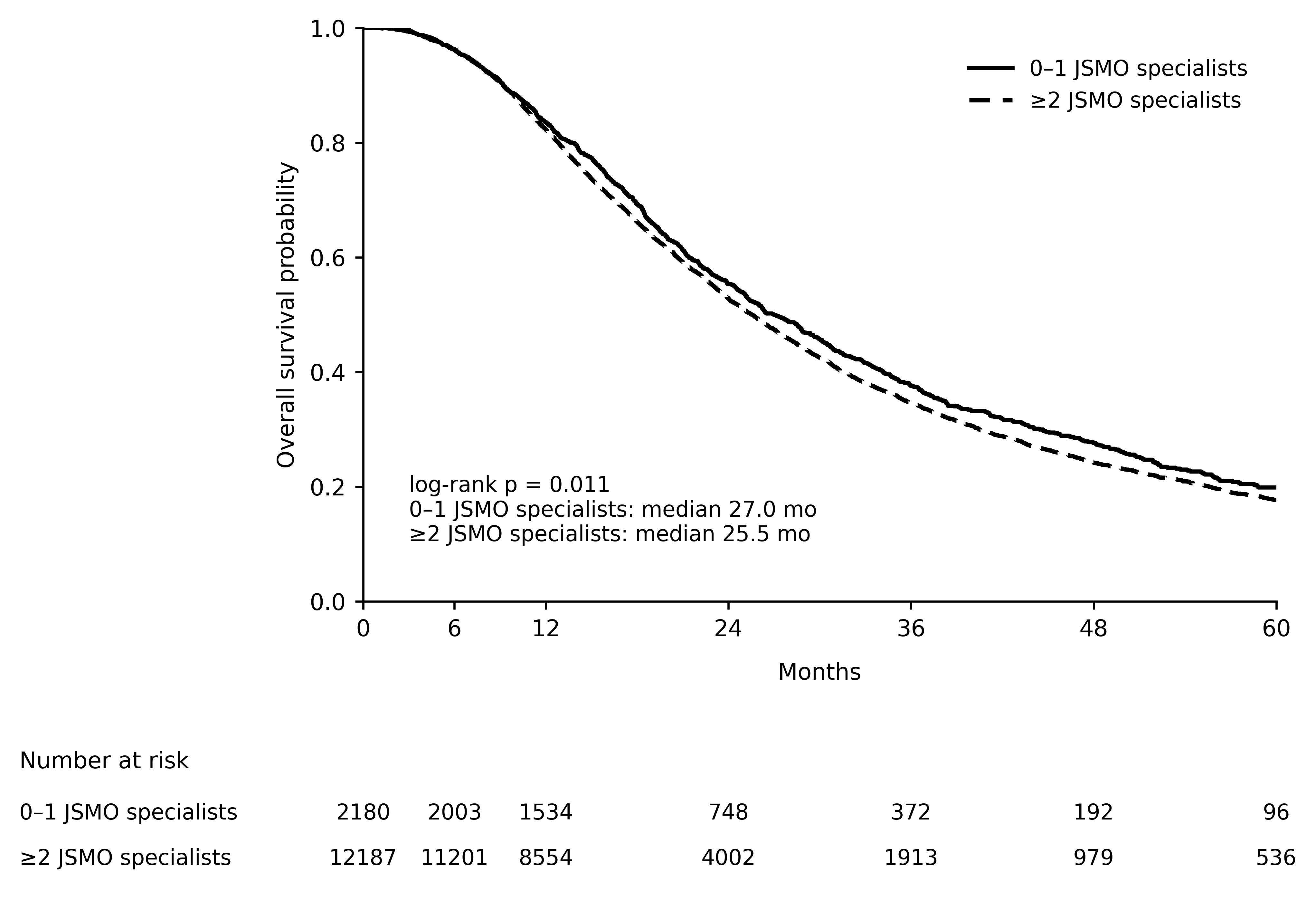

Supplement: Supplementary file 1 [file curroncol-33-00393-s001.zip › 37_CO_SFigure S2_supportive_OS_20260614.tiff]

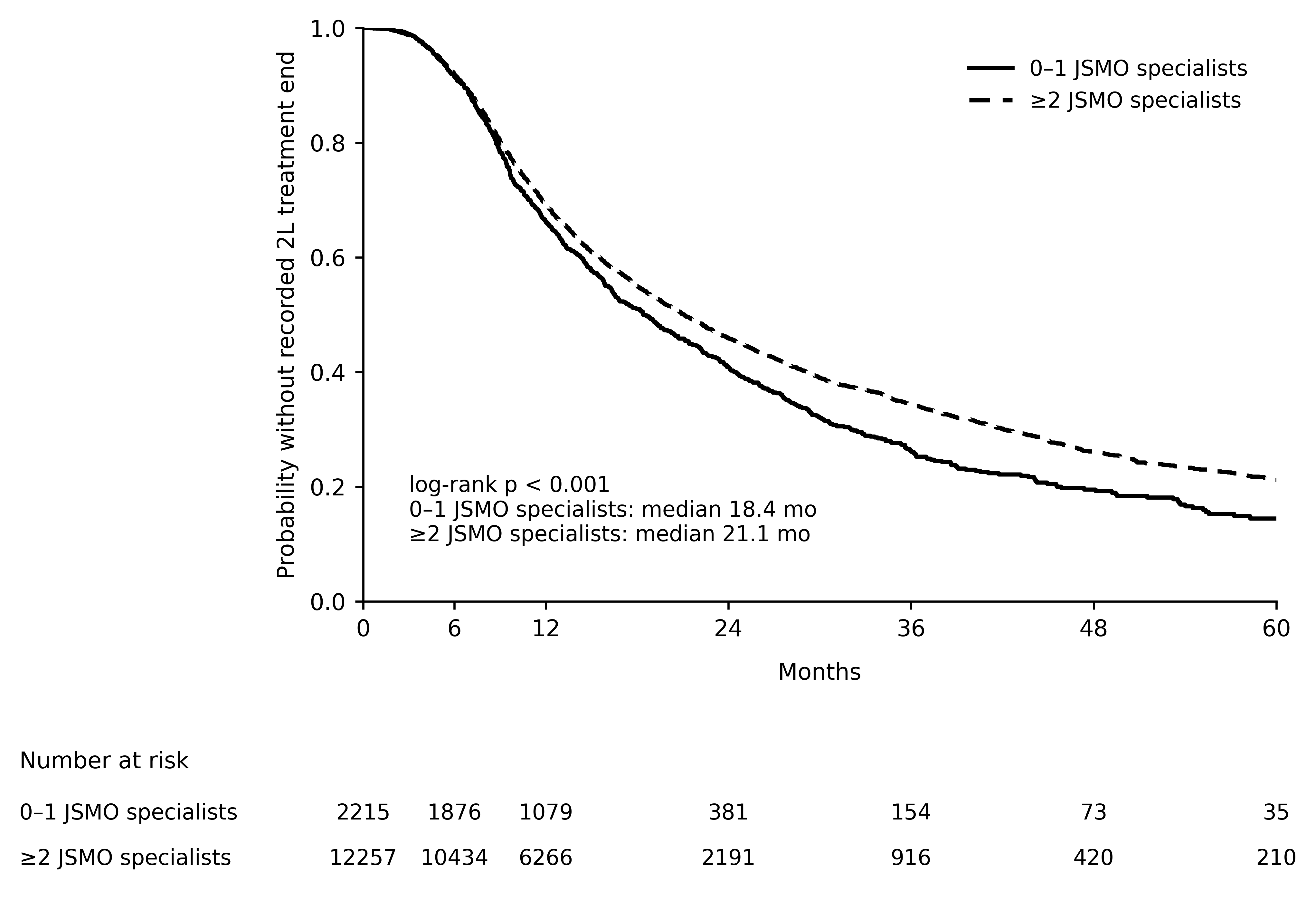

Supplement: Supplementary file 1 [file curroncol-33-00393-s001.zip › 37_CO_SFigure S3_secondary_2L_20260614.tiff]
